# The Influence of Gratitude on the Meaning of Life: The Mediating Effect of Family Function and Peer Relationship

**DOI:** 10.3389/fpsyg.2021.680795

**Published:** 2021-10-28

**Authors:** Ping Zhang, Lin Ye, Fang Fu, Lan Ge Zhang

**Affiliations:** ^1^Psychological Quality Education Center, Beijing University of Posts and Telecommunications, Beijing, China; ^2^College of Humanities and International Educational Exchange, Anhui University of Chinese Medicine, Hefei, China; ^3^School of Social Development and Public Policy, Fudan University, Shanghai, China

**Keywords:** gratitude, meaning existence, meaning seeking, family function, peer relationship

## Abstract

**Objective:** This study aimed to explore the influence of gratitude on the meaning of life of college students and the mediating effect of family function and peer relationships.

**Methods:** A total of 1,049 college students (mean age: 18.59 ± 0.96 years) were investigated by gratitude scale, family function scale, peer relationship questionnaire, and meaning of life scale.

**Results:** (1) Gratitude was positively correlated with meaning existence (*r* = 0.39, *P* < 0.05), meaning seeking (*r* = 0.23, *P* < 0.05), and peer relationship (*r* = 0.32, *P* < 0.05); meanwhile, it is also positively correlated with family function (*r* = 0.34, *P* < 0.05); (2) family function partially mediates the relationship between gratitude and meaning of life. Similarly, peer relationship partially mediates the relationship between gratitude and meaning of life.

**Conclusion:** The mediating model showed that grateful individuals can better sense the meaning of life by perceiving good family function and good peer relationships.

## Introduction

With the profound social transformation in China, the psychological problems of college students in the critical period of self-integration have become increasingly prominent. Among them, depression, anxiety, and even extreme behaviors such as self-injury and suicide of teenagers have become serious social problems (Gao et al., 2001; Li, 2013; Xu et al., 2021). At present, studies on the mental health of college students at home and abroad gradually focus on protective factors, such as enthusiasm (Kunter et al., 2011; Keller et al., 2016; Moè, 2016; Moè et al., 2020), enjoyment (Raccanello et al., 2019), resilience (Merrill et al., 2014; Tong, 2020), etc. Studies showed that improving protective factors can effectively prevent mental health problems (Henry et al., 2012) and help the healthy growth of youth (Magson et al., 2021). Since life meaning is a hot topic in the field of positive psychology in recent years, it is also a potential protective factor for the mental health of youth (Zhao et al., 2013; Xiu et al., 2019). Moreover, teenagers are in a critical period for gratitude development, self-pursuit, and self-affirmation (Li, 2013), and they seek to explore their world outlook, meaning, and life value (Yang and Wang, 2016), which has a profound impact on their lifelong development. Therefore, this study focuses on the influencing factors of the meaning of life of college students and its effect mechanism, so as to provide empirical support for further intervention strategies to help college students to grow in a positive manner.

### Meaning of Life and Gratitude

The meaning of life is a perception of an individual and awareness of the nature of human beings and their existence and of the more important things he/she thinks. It includes two aspects, namely, the presence of meaning and the search for meaning (Steger et al., 2006). Meaning existence is the cognitive dimension of life meaning, that is, to feel the own life and existence of an individual that is full of meaning, purpose, and reason. Meaning seeking is the motive dimension of the meaning of life, i.e., trying to find the reason for their existence or the meaning of life. The lack of life meaning leads to the lack of life goal, sense of existence, and sense of meaning and feeling empty, bored, and frustrated, which will affect the self-cognition and identification of an individual, leading to addictive behavior, anxiety, depression, and even increase in the risk of suicide (Fredrickson, 2004; Lin, 2016; Xu et al., 2019).

Recently, many scholars have focused on the relationship between gratitude and the meaning of life (Lin, 2019), and the research found that gratitude consistently predicted meaning in life (Kleiman et al., 2013). Gratitude is a mindful appreciation of benefits and gifts that can be attributed to the kindness of other people (McCullough et al., 2001). The influence of gratitude on the meaning of life of individuals is reflected in different levels. With respect to emotion, Fredrickson (2001) believed that “positive emotions broaden momentary thoughts and action repertoire, which in turn fuel personal relationship and psychological well-being.” Gratitude is a feeling of joy and appreciation and can be viewed as “part of a wider life orientation toward noticing and appreciating the positive in the world” (Wood et al., 2010). Individuals with this life orientation tend to experience more frequent, intense, and enduring associated states in daily life (Kleiman et al., 2013). Meanwhile, grateful individuals have stronger social connections and a more positive view of the social environment, so that they can get a stronger sense of belonging and connection (Lambert et al., 2010), which will acquire more opportunities to be aware of how meaningful their life is (Lin, 2019) as well as reinforce their tendency to maintain a meaningful life (Wood et al., 2009).

### Gratitude, Family Function, Peer Support, and Meaning of Life

According to find, remind, and bind theory (Algoe, 2012), gratitude, as a positive emotion, “updates our view of benefactor-specifically, finding new or reminding of current high-quality relationship partners, and helps to bind the people in the relationship more closely together.” Studies showed (Algoe et al., 2010) that gratitude promotes relationship-maintenance and relationship-strengthening behaviors across various types of relationships. Individuals with more gratitude feel closer to family members and peers (Algoe et al., 2010), meanwhile report more satisfaction with their relationships and engaging in more relationship-maintenance behaviors, such as trying to resolve conflict and expressing appreciation to each other (Kubacka et al., 2011). Moreover, gratitude has three morally relevant functions, namely, moral barometer function, moral motive function, and moral reinforcer function, which can strengthen their family and peer relationships (McCullough et al., 2001). For instance, Williams and Bartlett (2015) showed that the expression of gratitude facilitates affiliation in their relationships via friendliness, thoughtfulness, etc. Empirical studies also showed that gratitude could promote good social relationships (Tian et al., 2015). To be more specific, gratitude was found to be significantly associated with family function and peer support (Wang et al., 2011).

Family function refers to structural and organizational properties of the family group and the patterns of transactions among family members (Epstein et al., 1983), which is closely associated with the meaning of life. The healthier the family function is, the more possibility that teenagers might search for meaning with the help of family members (Wei et al., 2019). The more deficient the family function is, the less tendency teenagers might appreciate the sense of life meaning (Ping and Fang, 2017). To sum up, gratitude and family function are the sources of life meaning.

From the perspective of the interpersonal environment, family and peers are the important factors influencing the adaptation and development of adolescents (Xu et al., 2019). Generally speaking, youth values more on peer relationships, the influence of which even goes beyond the teacher-student relationship or even parent-child relationship. Gratitude emphasizes interpersonal psychological strength, including the appreciation of the benefits that have been obtained from others (Wood et al., 2008), and the expression of gratitude can help individuals to establish long-term interpersonal relationships (Hill et al., 2016).

In conclusion, grateful individuals tend to obtain closer social connections and promote a stronger sense of belonging and connection (Lambert et al., 2010), which in turn provide more opportunities for people to consider how meaningful their life is (Lin, 2019), strengthen their life goals, and reinforce their tendency to maintain a meaningful life (Wood et al., 2009).

### The Aim of This Study

Plenty of studies find support for the relationship between gratitude and the meaning of life; nevertheless, few empirical studies have been conducted on the mediating mechanism between gratitude and the meaning of life, and the exploration of the mediating role will help to elucidate how or why gratitude affects the meaning of life. Therefore, this study intends to investigate the influence of gratitude on the meaning of life of college students and further explores the mediating role of family function and peer relationship so as to provide more targeted empirical support for mental health education of college students.

**Figure 1:**
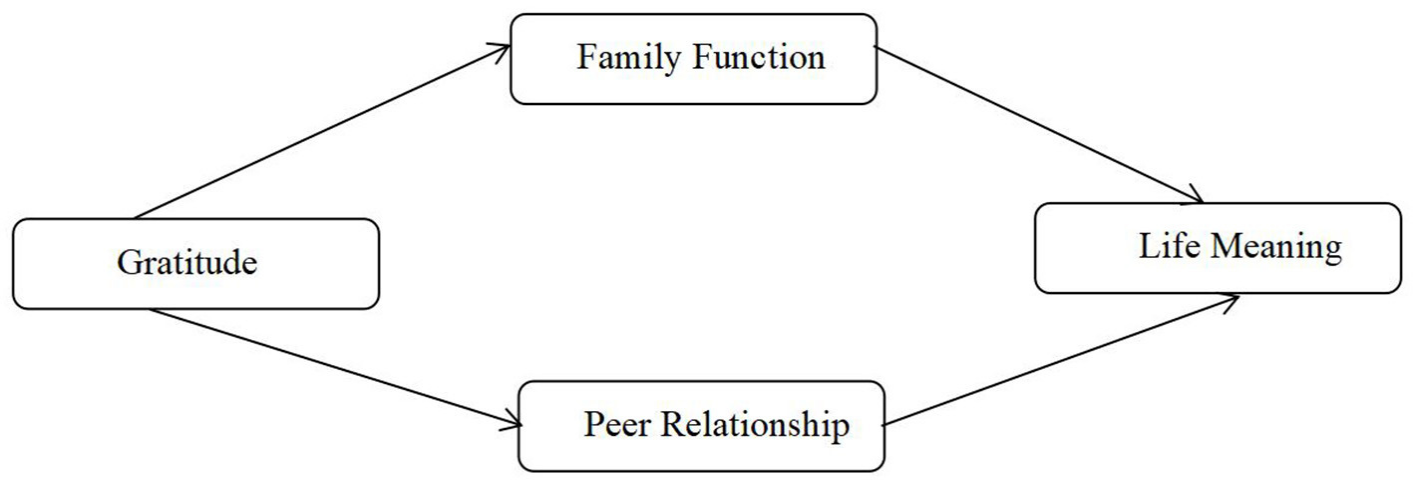
Theoretical model of the study.

To be more specific, this study examines the impact of gratitude on the meaning of life (meaning existence and meaning seeking) (Hypothesis 1) and the mediating role of family function and peer relationship quality in the impact of gratitude on the meaning of life (meaning existence and meaning seeking) (Hypotheses 2 and 3). From the perspective of positive psychology, in line with the broaden-and-build theory of gratitude, this study constructs the intermediary model of gratitude and the meaning of life (Figure 1), which provides theoretical support for gratitude stimulation and life education of youths.

## Methodology

### Subjects

A simple random cluster sampling test on college students was conducted in Beijing in January 2020. The minimum sample size is 10 times the total number of items of the questionnaire used in this study (Qiu, 2013). Since the scales in this study consist of 100 items, the sample size is estimated to be about 1,000, plus 20% of the missing value. Therefore, this study is expected to send out 1,200 questionnaires, and samples with more than 10% missing answers were excluded. We finally got 1,049 questionnaires, with an effective response rate of 87.42%. In this study, all the manipulations, measures, and analyses we did have been reported. There are 538 female students and 511 male students, with 762 freshmen (72.64%), 243 sophomores (23.16%), and 44 juniors (4.19%). The average age was 18.59 ± 0.96 years.

### Measurements

#### Grateful Disposition Questionnaire

The Gratitude Disposition Questionnaire (GQ-6) (Mc Cullough et al., 2002) was used in the Chinese validation by Chun et al. (2014). It contains six items to be rated from completely disagree to completely agree. In this study, the internal consistency coefficient of the scale is 0.71.

#### Family Function Scale

The Family Assessment Device (FAD) (Epstein et al., 1983) has 60 items and seven subscales. The subscale of general functioning (GF) consists of 12 items, which assesses the overall healthy/pathology of the family. Example items were “we can talk to each other about the sadness we feel,” “we feel accepted for what we are,” etc. (1 strongly disagree to 4 strongly agree). The higher the score was, the better the family function was. In this study, the internal consistency coefficient of the scale is 0.73.

#### Peer Relationship Questionnaire

The inventory of parent and peer attachment (IPPA) (Armsden and Greenberg, 1987) is designed to assess the relationship of teenagers with both their parents and peers. It consists of three forms for mother, father, and peer, each has 25 items with three dimensions, namely, alienation (e.g., I get upset a lot more than my friends knows about), communication (e.g., I tell my friends about my problems and troubles), and trust (e.g., I trust my friends). The dimension of alienation is reversely scored. The item format was a 5-point Likert scale from 1 (almost always or always true), 2 (often true), 3 (sometimes true), 4 (rarely true) to 5 (almost never or never true). The higher the score, the better the peer relationship. The internal consistency reliability of the scale was 0.89, and the internal consistency coefficient is 0.80.

#### Meaning of Life Scale

The meaning in life questionnaire (MLQ) (Steger et al., 2006) is a 7-point Likert scale, including two subscales of the presence of meaning (mlq-p) and the search for meaning (mlq-s). The scores of the two dimensions cannot be summed up, as they represent different contexts of the meaning of life. Therefore, in data processing, meaning existence and meaning seeking are regarded as two independent variables. In this study, the internal consistency coefficient of the scale is 0.81.

### Research Procedures

Students from several colleges and universities in Beijing were randomly selected for the test. The trained test administrators entered the class site to conduct the survey. The participants filled the questionnaires after reading and filling the informed consent form.

### Data Analysis

Data analysis was performed using IBM SPSS version 23.0 by USA, which includes the following three steps: (1) descriptive statistics of main variables and Pearson's correlation analysis; (2) hierarchical regression analysis is used to test Hypothesis 1; and (3) based on the process procedure of Model 4 for SPSS release 2.15 module (Hayes, 2013), the bootstrapping method was used to test the mediating role of family function and peer relationship in the relationship between gratitude and meaning of life (meaning existence and meaning seeking), that is, and two models were established to test Hypotheses 2 and 3.

## Results

### Common Method Bias Test

To reduce the common method bias, different scoring methods, response sentences, and answers were used during the test. The subjects were told to keep secret, and the questionnaire was only used for research. Before data processing, Harman single factor test was used. There are eight factors with eigenvalues >1, and the variation explained by the first factor is 22.35%, which is less than the critical criteria of 40%, indicating that the common method bias is not obvious.

### Descriptive Statistics and Correlation Analysis

The results in Table 1 showed that gratitude was moderately correlated with meaning existence (*r* = 0.39, *P* < 0.05), meaning seeking (*r* = 0.23, *P* < 0.05), and peer relationship (*r* = 0.32, *P* < 0.05), while moderately correlated with family function (*r* = 0.34, *P* < 0.05). Meanwhile, family function was moderately associated with meaning existence (*r* = 0.33, *P* < 0.05) and meaning seeking (*r* = 0.23, *P* < 0.05). In addition, there was a moderate positive correlation between peer relationship and meaning existence (*r* = 0.35, *P* < 0.05) and meaning seeking (*r* = 0.33, *P* < 0.05), while peer relationship and family function were also moderately correlated (*r* = 0.44, *P* < 0.05). Age and gender, which were weakly associated with some major variables, were controlled in this study.

**Table 1:** Descriptive statistics of main variables.

	***M***	***SD***	**1**	**2**	**3**	**4**	**5**
Gratitude	4.74	0.64	1				
Meaning existence	4.98	0.91	0.39^**^	1			
Meaning seeking	4.80	1.02	0.23^**^	0.63^**^	1		
Family function	2.03	0.40	0.34^**^	0.33^**^	0.23^**^	1	
Peer relationship	3.74	0.59	0.32^**^	0.35^**^	0.33^**^	−0.44^**^	1

** *P < 0.01*.

### Mediation Model Test

Hypothesis 1 (the direct relationship between gratitude and meaning existence and meaning seeking) was tested by using the hierarchical regression analysis. The results showed that after controlling for gender and age, gratitude had a significant effect on meaning existence and meaning seeking (*b* = 0.26, *P* < 0.001; *b* = 0.33, *P* < 0.001, respectively). Then, Hypotheses 2 and 3 (the simple mediating effect of family function and peer relationship) were tested by using the SPSS macro process, which employs the bootstrap method to test the significance of the indirect effect, that is, the indirect effect (*a* × *b*) of gratitude related to the meaning of life (meaning existence and meaning seeking) through the family. Model 4 was selected in PROCESS, and the sampling was set to 5,000 times, while the CI test level was set to 95%.

**Figure 2:**
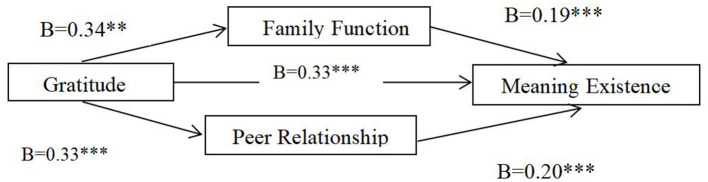
The mediating effect model of family function and peer relationship between gratitude and meaningful existence. **p < 0.01, ***p < 0.000.

As shown in Table 2, the indirect effect of gratitude on meaning existence through family function was significant (*a* × *b* = 0.26, SE = 0.03, and bootsci = [0.20, 0.32]); meanwhile, the indirect effect of gratitude on meaning existence through peer relationship was also significant (*a* × *b* = 0.12, SE = 0.02, and bootsci = [0.09, 0.15]), and the CI did not include 0. Therefore, family function and peer relationship play a mediating role between gratitude and meaningful existence (Figure 2).

**Table 2:** Results of PROCESS test.

**Independent variable**	**Dependent variable**								
	**Family function**			**Peer relationship**			**Meaning existence**		
	***Coeff*.**	***SE***	***P***	***Coeff*.**	***SE***	***P***	***Coeff*.**	***SE***	***P***
Controlled variable									
Gender	0.09	0.06	>0.05	−0.29	0.06	<0.001	0.10	0.06	>0.05
Predict variable									
Gratitude	0.34	0.03	<0.001	0.33	0.03	<0.001	0.26	0.03	<0.001
Family function	—	—	—	—	—	—	−0.15	0.03	<0.001
Peer relationship	—	—	—	—	—	—	0.20	0.03	<0.001
Constant	−1.09	0.56	<0.05	2.00	0.56	<0.01	−0.56	0.53	<0.05
*R* ^2^	0.11			0.12			0.22		
*F*	45.10^***^			47.48^***^			58.91^***^		

*** *p < 0.000*.

**Figure 3:**
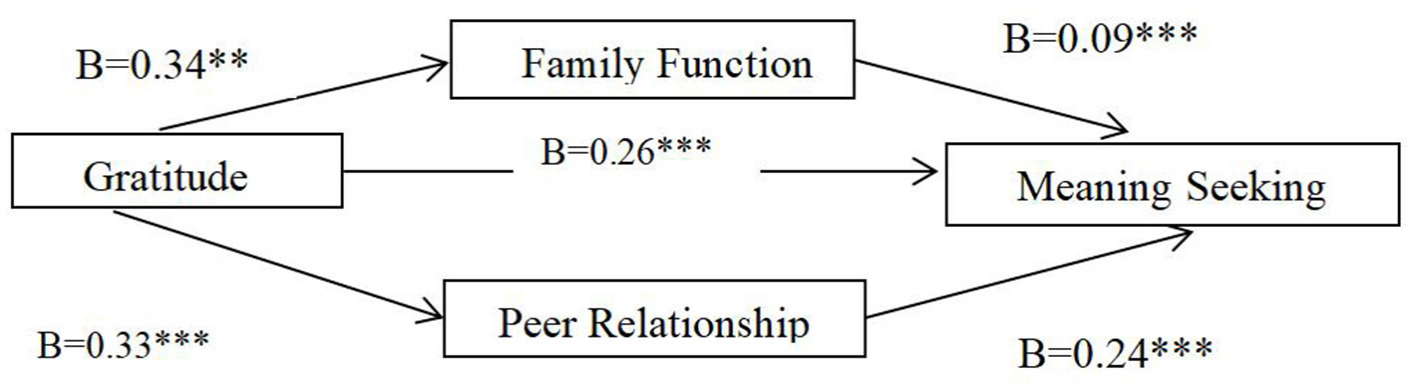
The mediating effect model of family function and peer relationship between gratitude and meaningful seeking. **p < 0.01, ***p < 0.000.

In addition, as shown in Table 3, the indirect effect of gratitude on meaning seeking through family function was significant (*a* × *b* = 0.03, SE = 0.01, and bootsci = [0.01, 0.06]), while the indirect effect of gratitude on meaning existence through peer relationship was also significant (*a* × *b* = 0.08, SE = 0.02, and bootsci = [0.05, 0.14]) and the CI did not include 0. Therefore, family function and peer relationship play a mediating role in the relationship between gratitude and revise meaningful existence to meaningful seeking (Figure 3).

**Table 3:** Results of PROCESS test 2.

**Independent variable**	**Dependent variable**								
	**Family function**			**Peer relationship**			**Meaning seeking**		
	***Coeff*.**	***SE***	***P***	***Coeff*.**	***SE***	***P***	***Coeff*.**	***SE***	***P***
Controlled variable									
Gender	0.09	0.06	>0.05	−0.29	0.06	<0.001	−0.08	0.06	>0.05
Predict variable									
Gratitude	−0.34	0.03	<0.001	0.33	0.03	<0.001	0.33	0.03	<0.001
Family function	—	—	—	—	—	—	−0.09	0.03	<0.05
Peer relationship	—	—	—	—	—	—	0.24	0.03	<0.001
Constant	−1.09	0.56	<0.001	2.00	0.56	<0.005	0.33	0.56	>0.05
*R* ^2^	0.11			0.12			0.13		
*F*	45.10^***^			47.48^***^			30.26^***^		

*** *p < 0.000*.

## Discussion

In the investigation of the mechanism of gratitude to the meaning of life, this study provides evidence that family function and peer relationship are critical and play an important role between these two variables. Our findings extend recent research on close relationships that cultivate a sense of thankfulness and gratitude for adolescents is vital for maintaining healthy family function and good peer relationships, which enable them to focus on the positive side of life and find life meaning.

### The Relationship Between Gratitude and the Meaning of Life

Previous studies showed that gratitude positively predicted the meaning of life (Lin, 2016). The results of this study further supported the fact that gratitude demonstrated a positive correlation with the meaning of life. This study found that people with more gratitude had more possibility to find their life meaning. Erickson believed that late adolescence is the key stage for individuals to seek identity, so benefiting from a healthy world outlook and emotion (e.g., through gratitude) will increase their positive evaluation of themselves, and they are more likely to appreciate the meaningfulness and value of life (Lin, 2019).

Meanwhile, the results of this study once again verify broaden and build function of gratitude. Highly grateful individuals pay more attention to positive experiences and positive emotions that contribute to the generation of meaning and promote individuals to feel and further seek the meaning of life. This suggests that educators should promote the generation and meaning seeking through positive gratitude education, so as to make individual life more meaningful. For instance, an effective method for enhancing gratitude can be shared with adolescents such as reflection on the three good things each day, recognizing three key aspects of helping behavior, and expressing gratitude toward others who help them (Baumsteiger et al., 2019).

### The Mediating Role of Family Function and Peer Relationship

The relationship between gratitude and the meaning of life has been verified by previous studies; nevertheless, it is not clear about the empirical link between gratitude and the meaning of life. The results of the mediating model showed that gratitude affects the meaning of life through the following two paths: first, gratitude affects the meaning of life through family function. Second, gratitude affects the meaning of life through peer relationships. Many studies provide evidence to support the central role of gratitude in promoting and maintaining high-quality relationships (Algoe et al., 2010; Kubacka et al., 2011). This study expands on these findings by depicting the path from gratitude to meaning of life through family function and peer relationships. On the one hand, gratitude involves the perception of others, which is a social and a pro-social incentive factor (Mc Cullough et al., 2008). Grateful individuals transform their emotional experience into helping others and feel grateful, which in turn results in a better perception of family function. Individuals with a higher gratitude tendency are more likely to express gratitude (Mc Cullough et al., 2008), which can also promote the construction and perception of good family relationships and family atmosphere. A good family environment, parenting style, and family atmosphere constantly nourish individuals and make them feel the meaning of life (Wei et al., 2019).

On the other hand, the results show that gratitude enhances the meaning of life through peer relationships. The perception of good peer relationships enables individuals to get more emotional support (Wang et al., 2014) so that individuals can perceive more abundant life meaning. This suggests that the more grateful the person is, the better he/she can feel the quality of peer relationships and the higher meaning of life he/she will feel.

Another notable finding is that the relationship of gratitude with peer relationships explained the relationship of gratitude to search for meaning more strongly than the relationship of gratitude with family function did. As Erikson (1966) postulated, adolescents were in the process of seeking self-identity and acquiring autonomy, and they may enjoy the company of peers rather than family members and explore their identity and life meaning together with them. Therefore, it is essential to provide platforms and opportunities for youths to discuss and find their role and life meaning together in school and communities.

In conclusion, the whole model implies the effect mechanism of gratitude on the meaning of life through family function and peer relationship, that is, the more individuals know how to be grateful, the more they know how to cherish the people and emotions around them. The more individuals feel grateful, the better they will treat others. Consequently, they will get more favor and support from others, which forms a virtuous circle. It is particularly important for young people to feel the beauty of the relationship. At this stage, individuals are very concerned about the perception of the relationship. With a good relationship foundation, they will feel that life is more meaningful. Gratitude can strengthen the relationship and promote the formation and maintenance of the relationship, as well as the connection and satisfaction of the relationship. Family and peer, as two essential environmental variables, provide more emotional support and social connection for individual development. They are also intimate resources in the youth relationship network, which are of great significance to individual life. Gratitude has a strong positive correlation with real-life, and its unique social characteristics seem to have adaptive value, which helps to promote the tendency of cooperation between individuals and non-family members (Mc Cullough et al., 2008) and maintain mutually beneficial altruism. Mc Cullough believed that gratitude, as the adaptability of altruistic behavior, plays a unique role in the evolution of human society (Algoe, 2012). This study found that gratitude can also promote good cooperation tendency between individuals and family members, increase their willingness to help others, sense the goodwill of others, and enhance the sense of the meaning of life. Individuals can get better evolution in the competition of natural selection, which is in line with the purpose of evolution and has evolutionary adaptability.

### Limitations and Future Research Directions

First, this study adopts a cross-sectional design, which cannot further judge the causal relationship among gratitude, family function, peer relationship, and meaning of life. Moreover, because the data are correlational, alternative relationships are not discussed in this study. For instance, individuals who have good family relationships become more grateful, which leads to increased meaning. Meanwhile, longitudinal research should be done to verify it. Second, gratitude and peer relationship have multiple dimensions, and the relationship between different dimensions with other variables may be different. Future research should pay attention to the examination of each dimension of these two variables to determine their different roles. Finally, this study includes only the sample of Chinese college students, and the generalization of the conclusion should be careful.

## Conclusion

This study enriches a positive emotion of gratitude model, broadens, and builds theory, and the research perspectives of cooperative evolution. The results provide guidance and intervention on how to improve the meaning of life of college students and provide important theoretical basis and method guidance for improving the mental health of youths. For instance, to improve the grateful disposition of adolescents, they can record five grateful things every week (Mc Cullough et al., 2004) or write gratitude letters to others (Seligman, 2002), as well as take part in gratitude group intervention (Sun and Chen, 2013). The more individuals know how to be grateful, the more they know how to cherish the people around them and consequently, they will get more favor and support from others as well as find their life meaning.

## Data Availability Statement

The raw data supporting the conclusions of this article will be made available by the authors, without undue reservation.

## Ethics Statement

The studies involving human participants were reviewed and approved by Beijing University of Posts and Telecommunications. The patients/participants provided their written informed consent to participate in this study.

## Author Contributions

PZ was mainly responsible for article conception, data collection, data analysis, and article writing. LY was mainly responsible for the article design, literature review, and article writing. FF was editing the paper and giving suggestions. LGZ was responsible for literature review. All authors contributed to the article and approved the submitted version.

## Conflict of Interest

The authors declare that the research was conducted in the absence of any commercial or financial relationships that could be construed as a potential conflict of interest.

## Publisher's Note

All claims expressed in this article are solely those of the authors and do not necessarily represent those of their affiliated organizations, or those of the publisher, the editors and the reviewers. Any product that may be evaluated in this article, or claim that may be made by its manufacturer, is not guaranteed or endorsed by the publisher.

## References

[B1] Algoe S. B. (2012). Find, remind and bind: the functions of gratitude in everyday relationships. Soc. Person. Psychol. Compass 6, 455–469. 10.1111/j.1751-9004.2012.00439.x

[B2] Algoe S. B. Gable S. L. Maisel N. C. (2010). It's the little things: everyday gratitude as a booster shot for romantic relationships. Person. Relat. 17, 217–233. 10.1111/j.1475-6811.2010.01273.x

[B3] Armsden G. C. Greenberg M. T. (1987). The inventory of parent and peer attachment: individual differences and their relationship to psychological well-being in adolescence. J. Youth Adolesc. 16, 427–454. 10.1007/BF02202939 24277469

[B4] Baumsteiger R. Mangan S. Bronk K. C. Bono G. (2019). An integrative intervention for cultivating gratitude among adolescents and young adults. J. Positive Psychol. 46, 1–13. 10.1080/17439760.2019.1579356

[B5] Chun X. W. Xiao Z. Lin L. L. Lin J. C. (2014). The effect of gratitude on posttraumatic growth in adolescents: the mediating role of social support and active rumination. J. Psychol. Sci. 37, 1148–1153. http://CNKI:SUN:XLFZ.0.2014-01-010

[B6] Epstein N. B. Baldwin L. M. Bishop D. S. (1983). The mcmaster family assessment device. J. Marital Fam. Ther. 9, 171–180. 10.1111/j.1752-0606.1983.tb01497.x

[B7] Erikson E. H. (1966). Eight ages of man. Int. J. Psychiatry 2:281. 5934808

[B8] Fredrickson B. L. (2001). The role of positive emotions in positive psychology: the broaden - and - build theory of positive emotions. Am. Psychol. 56, 218–226. 10.1037/0003-066X.56.3.218 11315248 PMC3122271

[B9] Fredrickson B. L. (2004). Gratitude, like other positive emotions, broadens and builds, in The Psychology of Gratitude, eds Emmons R. A. Mc Cullough M. E. (New York, NY: Oxford University Press), 145–166. 10.1093/acprof:oso/9780195150100.003.0008

[B10] Gao M. Zhang J. X. Xu F. Shen Q. F. (2001). Depression, anxiety and related factors in adolescents. Anhui J. Prevent. Med. 4, 250–251.

[B11] Hayes A. F. (2013). Introduction to mediation, moderation, and conditional process analysis: a regression- based approach. J. Educ. Meas. 51, 335–337. 10.1111/jedm.12050

[B12] Henry D. Allen J. Fok C. C. Rasmus S. Charles B. (2012). Patterns of protective factors in an intervention for the prevention of suicide and alcohol abuse with Yupik Alaska Native Youth. Am. J. Drug Alcohol Abuse 38, 476–482. 10.3109/00952990.2012.704460 22931082 PMC3506184

[B13] Hill R. L. Edmonds G. W. Peterson M. Luyckx K. Andrews J. A. (2016). Purpose in life in emerging adulthood: development and validation of a new brief measure. J. Positive Psychol. 11, 1–9. 10.1080/17439760.2015.1048817 26958072 PMC4779362

[B14] Keller M. M. Hoy W. A. Goetz T. Frenzel A. C. (2016). Teacher enthusiasm: reviewing and redefining a complex construct. Educ. Psychol. Rev. 28, 743–769. 10.1007/s10648-015-9354-y

[B15] Kleiman E. M. Adams L. M. Kashdan T. B. Riskind J. H. (2013). Gratitude and grit indirectly reduce risk of suicidal ideations by enhancing meaning in life: evidence for a mediated moderation. J. Res. Pers. 5, 539–546. 10.1016/j.jrp.2013.04.007

[B16] Kubacka K. E. Finkenauer C. Rusbult C. E. Keijsers L. (2011). Maintaining close relationships: gratitude as a motivator and a detector of maintenance behavior. Person. Soc. Psychol. Bull. 37, 1362–1375. 10.1177/0146167211412196 21670220

[B17] Kunter M. Frenzel A. Nagy G. Baumert J. Pekrun R. (2011). Teacher enthusiasm:Dimensionality and context specificity. Contemp. Educ. Psychol. 36, 289–301. 10.1016/j.cedpsych.2011.07.001

[B18] Lambert N. M. Clark M. S. Durtschi J. Fincham F. D. Graham S. M. (2010). Benefits of expressing gratitude: expressing gratitude to a partner changes one's view of the relationship. Psychol. Sci. 21, 574–580. 10.1177/0956797610364003 20424104

[B19] Li Y. X. (2013). Depression, anxiety and related factors in adolescents. Chin. J. Health Psychol. 21, 415–417.

[B20] Lin C. C. (2019). Gratitude and suicidal ideation in undergraduates in Taiwan: the mediating role of self-esteem and meaning in life. J. Death Dying 0, 1–17. 10.1177/0030222819882845 31623525

[B21] Lin X. J. (2016). The study of the education of the life meaning of youth from the perspective of social control. Psychol. Explorat. 36, 392–396. 10.3969/j.issn.1003-5184.2016.05.002

[B22] Magson N. R. Freeman J. Rapee R. M. Richardson C. E. Fardouly J. (2021). Risk and protective factors for prospective changes in adolescent mental health during the covid-19 pandemic. J. Youth Adolesc. 50, 44–57. 10.1007/s10964-020-01332-9 33108542 PMC7590912

[B23] Mc Cullough M. E. Emmon R. A. Tsang J. A. (2002). The grateful disposition: a conceptual and empirical topography. J. Person. Soc. Psychol. 1, 112–127. 10.1037/0022-3514.82.1.112 11811629

[B24] Mc Cullough M. E. Kimeldorf M. B. Cohen A. D. (2008). An adaptation for altruism? The social causes, social effects, and social evolution of gratitude. Curr. Direct. Psychol. Sci. 17, 281–284. 10.1111/j.1467-8721.2008.00590.x

[B25] Mc Cullough M. E. Tsang J. Emmons R. A. (2004). Gratitude intermediate affective terrain: links of grateful moods with individual differences and daily, emotional experience. J. Person. Soc. Psychol. 86, 295–309. 10.1037/0022-3514.86.2.295 14769085

[B26] McCullough M. E. Kilpatrick S. D. Emmons R. A. Larson D. B. (2001). Is gratitude a moral affect? Psychol. Bull. 127, 249–266. 10.1037/0033-2909.127.2.249 11316013

[B27] Merrill W. S. Ware N. Hakizimana L. Tugenberg T. Currie M. Dahnweih G.. (2014). Fostering resilience: protective agents, resources, and mechanisms for adolescent refugees' psychosocial well-being. Adolesc. Psychiatry 4, 164–176. 10.2174/221067660403140912162410 25544939 PMC4274391

[B28] Moè A. (2016). Does displayed enthusiasm favour recall, intrinsic motivation and time estimation? Cogn. Emot. 30, 1361–1369. 10.1080/02699931.2015.1061480 26211683

[B29] Moè A. Frenzel A. C. Au L. Taxer J. L. (2020). Displayed enthusiasm attracts attention and improves recall. Br. J. Educ. Psychol. 91, 911–927. 10.1111/bjep.12399 33325548

[B30] Ping Z. Fang L. (2017). Risk factors of parenting behavior among suicidal college students. Chin. J. Sch. Health 11, 1746–1749. 10.16835/j.cnki.1000-9817.2017.11.044

[B31] Qiu H. Z. (2013). Quantitative Analysis and Statistical Research. Chongqing: Chongqing University Press.

[B32] Raccanello D. Brondino M. Moè A. Stupnisky R. Lichtenfeld S. (2019). Enjoyment, boredom, anxiety in elementary schools in two domains: relations with achievement. J. Exp. Educ. 87, 449–469. 10.1080/00220973.2018.1448747

[B33] Seligman M. E. P. (eds). (2002). Authentic Happiness: Using the New Positive Psychology to Realize Your Potential for Lasting Fulfillment. New York, NY: Simon and Schuster.

[B34] Steger M. Frazier P. Oishi S. (2006). The meaning in life questionnaire: assessing the presence of and search for meaning in life. J. Couns. Psychol. 53, 80–93. 10.1037/0022-0167.53.1.80

[B35] Sun W. W. Chen M. F. (2013). Literature analysis and enlightenment of youth gratitude education. J. Yulin Univ. 0, 94–99. 10.3969/j.issn.008-3871.2013.06.026

[B36] Tian L. Du M. Huebner E. S. (2015). The effect of gratitude on elementary school students' subjective well-being in schools: the mediating role of prosocial behavior. Soc. Indicators Research 122, 887–904. 10.1007/s11205-014-0712-9

[B37] Tong X. L. (2020). Intervention on resilience model of high risk adolescents – Based on qualitative analysis of children in Northwest China? Soc. Sci. Res. 5, 130–138. Available online at: http://www.cnki.com.cn/Article/CJFDTotal-SHYJ202005017.htm

[B38] Wang J. P. Yu C. F. Zeng Y. Y. Ye T. Zhang W. (2011). The influencing factors and mechanism of adolescents' gratitude. Psycholo. Dev. Educ. 27, 260–266.

[B39] Wang L. J. Wang W. C. Gu H. G. (2014). Relationships among teacher support peer conflict resolution and school emotional experiences in adolescents from Shanghai. Soc. Behav. Person. 42, 99–113. Available online at: https://xueshu.baidu.com/usercenter/paper/show?paperid=17570gj008760650en6k0aa0ea752543&site=xueshu_se-2021-09-30

[B40] Wei J. C. Dong R. Y. Di K. G. Shi G. N. (2019). The promotion of family function to the hope of Higher Vocational College Students: The mediating role between the existence of life meaning and search for meaning. Chin. J. Clin. Psychol. 27, 577–581. 10.16128/j.cnki.1005-3611.2019.03.030

[B41] Williams L. A. Bartlett M. Y. (2015). Warm thanks: gratitude express on facilitates social affiliation in new relationships via perceived warmth. Emotion 15, 1–5. 10.1037/emo0000017 25111881

[B42] Wood A. M. Froh J. J. Geraghty A. W. (2010). Gratitude and well-being: a review and theoretical integration. Clin. Psychol. Rev. 30, 890–905. 10.1016/j.cpr.2010.03.005 20451313

[B43] Wood A. M. Joseph S. Maltby J. (2008). Gratitude uniquely predicts satisfaction with life: incremental validity above the domains and facets of the five factor model. Pers. Individ. Dif. 45, 49–54. 10.1016/j.paid.2008.02.019

[B44] Wood A. M. Joseph S. Maltby J. (2009). Gratitude predicts psychological well-being above the big five facets. Pers. Individ. Dif. 46, 443–447. 10.1016/j.paid.2008.11.012

[B45] Xiu G. Z. Jie Q. Yu W. H. (2019). The relationship between sense of meaning and addiction tendency to mobile phone: the mediating role of self-control. Stud. Psychol. Behav. 17, 536–545. 10.3969/j.issn.1672-0628.2019.04.013

[B46] Xu X. Z. Ying H. G. Hua D. L. (2019). A study of the relationship between parent-child, peer, teacher-student relationship and subjective well-being of adolescent. Psychol. Dev. Educ. 35, 458–466. 10.16187/j.cnki.issn1001-4918.2019.04.09

[B47] Xu Y. Weng S. Y. Liu S. K. Jiang M. L. Yu M. Chen C. W. (2021). Investigation on the status quo of suicidal behavior and analysis of its influencing factors among adolescents in Guangdong Province. Shanghai Prevent Med. Available online at: http://kns.cnki.net/kcms/31.1635.R.20210518.1545.002.html (accessed May 27, 2021).

[B48] Yang Y. Q. Wang W. (2016). A review of the research on the meaning of youth's life. Sci. Educ. Collect. 1, 18–19.

[B49] Zhao J. B. Jiang M. J. Zhao J. B. Xiao R. Yang X. L. (2013). The moderating and mediating effects of sense of meaning of life on College Students' optimism and suicide risk. Chin. J. Behav. Med. Brain Sci. 22, 542–544.

